# Multiomic analysis on human cell model of wolfram syndrome reveals changes in mitochondrial morphology and function

**DOI:** 10.1186/s12964-021-00791-2

**Published:** 2021-11-20

**Authors:** Agnieszka Zmyslowska, Miljan Kuljanin, Beata Malachowska, Marcin Stanczak, Dominika Michalek, Aneta Wlodarczyk, Dagmara Grot, Joanna Taha, Bartłomiej Pawlik, Magdalena Lebiedzińska-Arciszewska, Hanna Nieznanska, Mariusz R. Wieckowski, Piotr Rieske, Joseph D. Mancias, Maciej Borowiec, Wojciech Mlynarski, Wojciech Fendler

**Affiliations:** 1 Department of Clinical Genetics, Medical University of Lodz, Pomorska Str. 251, 92-213 Lodz, Poland; 2 Department of Radiation Oncology, Dana-Farber Cancer Institute, Boston, MA USA; 3 Department of Biostatistics and Translational Medicine, Medical University of Lodz, Lodz, Poland; 4 Department of Radiation Oncology, Einstein College of Medicine, Bronx, NY USA; 5 Department of Tumor Biology, Medical University of Lodz, Lodz, Poland; 6 Central Laboratory for Genetic Research in Pediatric Oncology “Oncolab”, Medical University of Lodz, Lodz, Poland; 7 Department of Pediatrics, Oncology and Hematology, Medical University of Lodz, Lodz, Poland; 8 Postgraduate School of Molecular Medicine, Medical University of Warsaw, Warsaw, Poland; 9 Nencki Institute of Experimental Biology, Polish Academy of Sciences, Warsaw, Poland

**Keywords:** Wolfram syndrome, Proteomics, Transcriptomics, Mitochondria, ER stress

## Abstract

**Background:**

Wolfram syndrome (WFS) is a rare autosomal recessive syndrome in which diabetes mellitus and neurodegenerative disorders occur as a result of Wolframin deficiency and increased ER stress. In addition, WFS1 deficiency leads to calcium homeostasis disturbances and can change mitochondrial dynamics. The aim of this study was to evaluate protein levels and changes in gene transcription on human WFS cell model under experimental ER stress.

**Methods:**

We performed transcriptomic and proteomic analysis on WFS human cell model—skin fibroblasts reprogrammed into induced pluripotent stem (iPS) cells and then into neural stem cells (NSC) with subsequent ER stress induction using tunicamycin (TM). Results were cross-referenced with publicly available RNA sequencing data in hippocampi and hypothalami of mice with *WFS1* deficiency.

**Results:**

Proteomic analysis identified specific signal pathways that differ in NSC WFS cells from healthy ones. Next, detailed analysis of the proteins involved in the mitochondrial function showed the down-regulation of subunits of the respiratory chain complexes in NSC WFS cells, as well as the up-regulation of proteins involved in Krebs cycle and glycolysis when compared to the control cells. Based on pathway enrichment analysis we concluded that in samples from mice hippocampi the mitochondrial protein import machinery and OXPHOS were significantly down-regulated.

**Conclusions:**

Our results show the functional and morphological secondary mitochondrial damage in patients with WFS.

**Graphical Abstract:**

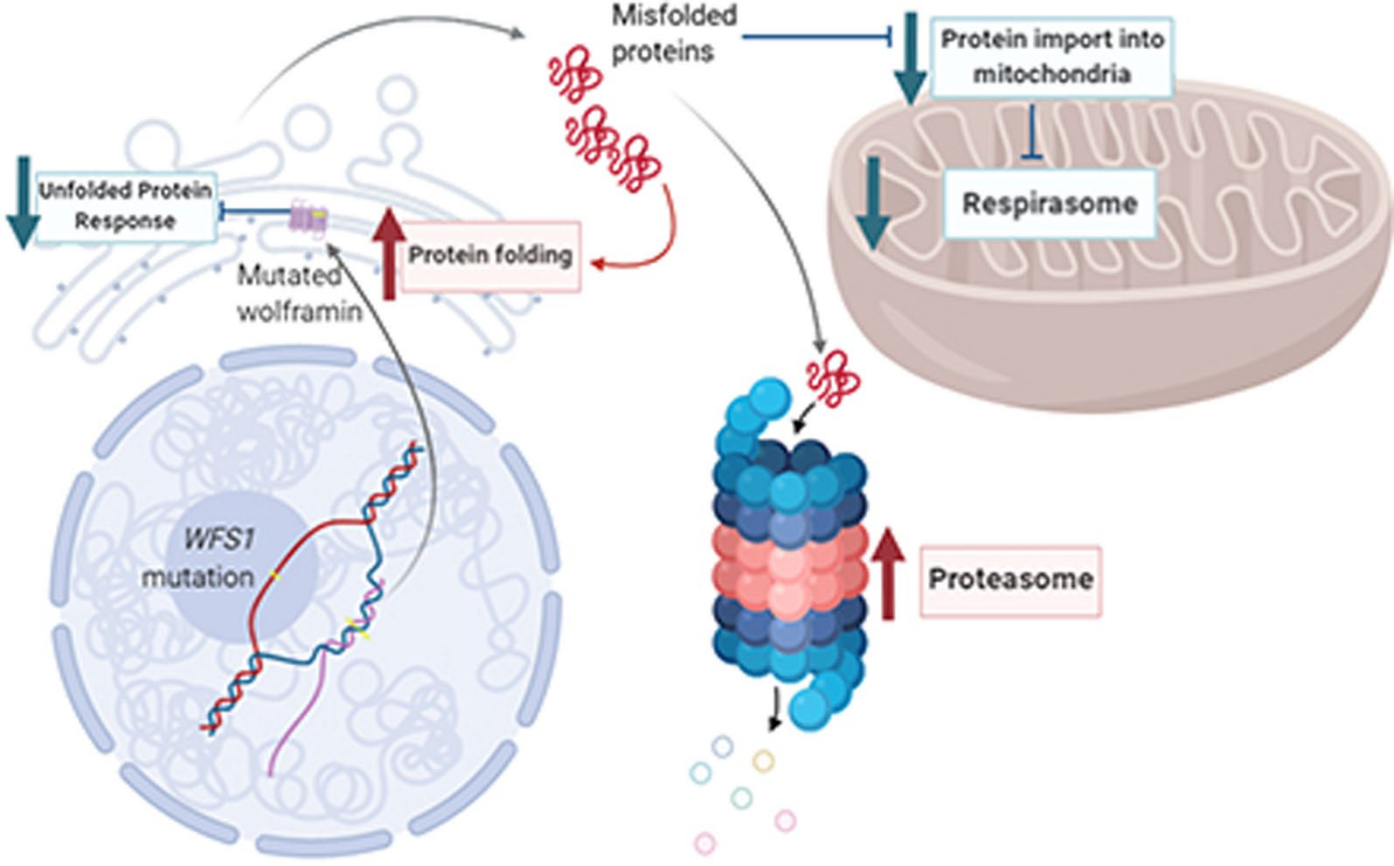

**Video Abstract**

**Supplementary Information:**

The online version contains supplementary material available at 10.1186/s12964-021-00791-2.

## Background

Wolfram syndrome (WFS) is a rare genetic syndrome inherited in an autosomal recessive manner, which occurs as a result of the presence of pathogenic variants mainly in the *WFS1* gene [1, 2]. In all patients with WFS many disorders are observed including diabetes mellitus, optic atrophy and progressive neurodegeneration, mainly involving the hippocampus, hypothalamus, cerebellum and brainstem [3]. Wolframin—the product of *WFS1* gene—is an integral component of the endoplasmic reticulum (ER) and is expressed in many organs (e.g., brain, pancreas, liver, heart). This protein plays a protective role against ER stress in affected cells and its functional lack in this syndrome results in a number of biological effects and finally, a wide range of clinical symptoms observed in WFS patients [4, 5].

The significance of ER stress in WFS has been already described. Once the ER has exceeded its folding capacity, accumulation of unfolded protein inside the ER lumen occurs. This results in ER stress which activates the signaling pathways associated with the unfolded protein response (UPR) [6]. It is known that in the absence of Wolframin, ER stress is increased by activation of transcription of both PERK kinase (Protein kinase RNA-like ER kinase) and ATF6 factor (Activating transcription factor 6). Moreover, as a result of the stimulation by requiring inositol factor 1 (IRE1), an unconventional splicing of the mRNA encoding the X-box binding protein 1 (XBP1) is observed. Activation of all three ER stress pathways results in increase in specific ER stress markers and apoptosis of the affected cells [7–10]. Furthermore, *WFS1* deficiency inducing ER stress leads to impaired cytosolic Ca^2+^ homeostasis and altered mitochondrial dynamics, as already suggested in an animal model of WFS and fibroblasts obtained from WFS patients [11, 12].

To date, no effective treatment is available for WFS patients [13, 14]. Attempting to explain the abnormalities observed in WFS at the protein level and assessing changes in gene transcription under experimental ER stress may provide an opportunity to precisely clarify pathological mechanisms and thus exploit the potential treatment.

In the present study, proteomic and transcriptomic analyses of a human cellular model of WFS was performed.

## Materials and methods

Diagnosis of WFS was confirmed in patients by direct sequencing of the *WFS1* gene and/or multiplex ligation-dependent probe amplification (MLPA; SALSA MLPA P163 GJB-WFS1 probemix, MRC-Holland, The Netherlands), as described previously [15]. Skin biopsies in WFS patients (n = 2 with nonsense mutations) and healthy volunteers (n = 2) were performed.

Next step included reprogramming process of skin fibroblasts to induced pluripotent stem (iPS) cells and then to trans-differentiated neural stem cells (NSC) with following induction of ER stress. This allowed us to create an experimental human cell model of WFS with enhanced ER stress effect.

### Cell cultures

Fibroblasts collected from skin biopsy were transformed into iPS and NSC in cooperation with Celther Company, Poland. Next, iPS cells were cultured in Essential 8 (Life Technologies, CA, USA) on protein-coated culture vessels of the extracellular matrix (Geltrex™ LDEV-Free Reduced Growth Factor Basement Membrane Matrix Geltrex, Life Technologies, CA, USA; 1:100). After reaching the appropriate confluence, iPS cell colonies were passed using 0.5 mM EDTA (Sigma-Aldrich, Germany) on new culture vessels and also extracellular matrix protein-coated, until the number of cells required for the analysis was reached.

The NSC were cultured in PSC Neural Induction Medium (Life Technologies, CA, USA) on protein-coated culture vessels of the extracellular matrix (Geltrex™ LDEV-Free Reduced Growth Factor Basement Membrane Matrix Geltrex, Life Technologies, CA, US; 1:100). After reaching appropriate confluence, the cells passed using StemPro Accutase (Life Technologies, CA, USA) on new culture vessels and also extracellular matrix protein-coated, until the number of cells required for the analysis was reached. Marker analysis showed that the cells obtained were NSC and then differentiation of these cells was performed, as described previously [16].

Briefly, before differentiation, cells were checked if they had SOX-2 and nestin, but if they were OCT-4 negative. Then NSC differentiation was performed. After 7 days of differentiation, almost 25% of cells showed MAP2 expression and neuronal morphology. Neither tyrosine hydroxylase (TH) cells nor GFAP cells were detected within these cells. Culture of NSCs for 14 days in differentiation medium did not result in any GFAP-positive cells, while single cells expressing TH were observed. Furthermore, the cells significantly changed their morphology and most of them, with long axons and dendrites, started to form clusters arranged in dense networks. After four weeks of differentiation, about 20% of cells became GFAP positive and astrocytic in morphology.

### ER stress induction

The cells were treated with ER stress inductor tunicamycin (TM, Sigma-Aldrich, Germany; 5 µg/ml) dissolved in DMSO (Sigma-Aldrich, Germany). The cells were incubated with TM and under control conditions (culture medium). After 8 h of incubation, the medium was collected from both culture vessels and centrifuged (200×*g*, 2 min). After removing the supernatant, the remaining cells were rinsed with PBS solution and then peeled off from the vessel surface. The cells were centrifuged (200×*g*, 5 min) and counted and then aliquoted for further analyses.

### Proteomic analysis

The NSC were then prepared for proteomic analysis, i.e. dry pellet of 10 million cells. For this purpose, an appropriate number of cells were washed twice with cold buffered saline solution without ions (PBS w/o Ca^2+^, Mg^2+^; vWR, PA, USA), inundated with a volume of cold PBS appropriate for the surface of the culture vessel, scraped and centrifuged (330×*g*, 5 min, 4 °C). After supernatant removal, the cells were quickly frozen in liquid nitrogen and then stored at − 80 °C. The whole procedure was performed on ice.

### Proteomics—materials

Isobaric TMT reagents and the BCA protein concentration assay kit were from ThermoFisher Scientific (Rockford, IL, USA). Empore-C18 material for in-house made Stage Tips was from 3 M (Saint Paul, MN, USA). Sep-Pak cartridges (100 mg size) were purchased from Waters (Milford, MA, USA). All solvents used for Liquid chromatography (LC) were purchased from J.T. Baker (Central Valley, PA, USA). Mass spectrometry (MS)-grade trypsin and Lys-C protease were purchased from ThermoFisher Scientific and Wako (Boston, MA, USA), respectively. Complete protease inhibitors were from Millipore Sigma (Saint Louis, MO, USA). Unless otherwise noted, all other chemicals were purchased from ThermoFisher Scientific.

### MS sample processing

All proteomic analyses for each patient and control samples were performed in triplicates. Cell pellets were lysed using 8 M urea, 200 mM 4-(2-hydroxyethyl)-1-piperazinepropanesulfonic acid (EPPS) at pH 8.5 with protease inhibitors (one tablet per 10 mL of lysis buffer. Samples were further homogenized and DNA was sheared via sonication using a probe sonicator (20 × 0.5 s pulses; level 3). Total protein was determined using a BCA assay and proteins were stored at − 80 °C until future use. A total of 25 μg of protein was aliquoted for each condition and TMT channel for further downstream processing. Protein extracts were reduced using 5 mM tris-(2-carboxyethyl) phosphine (TCEP) for 15 min at room temperature. Next, samples were alkylated with 10 mM iodoacetamide for 30 min in the dark at room temperature. To facilitate the removal of incompatible reagents, proteins were precipitated using chloroform and methanol. Briefly, to 100 μL of each sample, 400 μL of methanol was added, followed by 100 μL of chloroform with thorough vortexing. Next, 300 μL of HPLC grade water was added and samples were vortexed thoroughly. Each sample was centrifuged at 14,000×*g* for 5 min at room temperature. The upper aqueous layer was removed and the protein pellet was washed twice with methanol and centrifuged at 14,000×*g* for 5 min at room temperature. Protein pellets were re-solubilized in 200 mM EPPS buffer and digested overnight with Lys-C (1:100, enzyme:protein ratio) at room temperature. The next day, trypsin (1:100 ratio) was added and incubated at 37 °C for an additional 6 h in a ThermoMixer set to 1000 RPM.

### TMT labeling

To each digested sample, 30% anhydrous acetonitrile was added and 25 μg of peptides were labeled using ~ 55 μg of TMTPro reagents (TMT1-TMT16) for 1 h at room temperature with constant agitation. Following labeling, 5% hydroxylamine was added to quench excess TMT reagent. To equalize protein loading a ratio check was performed by pooling ~ 2 μg of each TMT-labeled sample. Samples were pooled and desalted using an in-house packed C18 Stage Tip and analyzed by liquid chromatography (LC) tandem mass spectrometry (MS/MS). Normalization factors derived from the ratio check were used to pool samples 1:1 across all TMT channels and the combined sample was desalted using a 100 mg Sep-Pak solid phase extraction cartridge. Eluted peptides were further fractionated using basic-pH reversed-phase (bRP) on an Agilent 300 extend C18 column and were collected into a 96 deep-well plate. Samples were consolidated into 24 fractions as previously described, and 12 nonadjacent fractions were desalted using Stage Tips prior to analyses using LC–MS/MS [17–19].

### Mass spectrometry and data acquisition

All mass spectrometry data were acquired using an Orbitrap Fusion Lumos mass spectrometer in-line with a Proxeon nanoLC-1200 Ultra performance LC (UPLC) system. TMT labeled peptides were separated using an in-house packed 100 µm capillary column with 35 cm of Accucore 150 resin (2.6 μm, 150 Å) (ThermoFisher Scientific) using either a 120 min LC gradient from 4 to 24% acetonitrile in 0.125% formic acid per run. Eluted peptides were acquired using synchronous precursor selection (SPS-MS3) method for TMT quantification. Briefly, MS1 spectra were acquired at 120 K resolving power with a maximum of 50 ms ion injection in the Orbitrap. MS2 spectra were acquired by selecting the top 10 most abundant features via collisional induced dissociation (CID) in the ion trap using an automatic gain control (AGC) of 15 K, quadrupole isolation width of 0.5 m/z and a maximum ion time of 50 ms. These spectra were passed in real time to the external computer for database searching. Intelligent data acquisition (IDA) using real-time searching (RTS) was performed using Orbiter as previously described [20, 21]. Peptide spectral matches were analyzed using the Comet search algorithm designed for spectral acquisition speed [22, 23]. Real-time access to spectral data was enabled by the ThermoFisher Scientific Fusion API. Briefly, peptides were filtered using simple filters that included the following: not a match to a reversed sequence, maximum PPM error 50, minimum XCorr of 0.5, minimum deltaCorr of 0.10 and minimum peptide length of 7. If peptide spectra matched to above criteria, an SPS-MS3 scan was performed using up to 10 *b-* and *y-type* fragment ions as precursors with an AGC of 200 K for a maximum of 200 ms with a normalized collision energy setting of 45.

### Mass spectrometry data analysis

All acquired data were searched using the open-source Comet algorithm using a previously described informatics pipeline [24–26]. We acknowledge Dr. Steven Gygi for use of a custom CORE data analysis software as part of the pipeline. Briefly, peptide spectral libraries were first filtered to a peptide false discovery rate (FDR) of less than 1% using linear discriminant analysis employing a target-decoy strategy. Spectral searches were done using a custom fasta formatted database which included common contaminants, reversed sequences with the following parameters: 50 PPM precursor tolerance, fully tryptic peptides, fragment ion tolerance of 0.9 Da and a static modification by TMT (+ 304.2071 Da) on lysine and peptide N termini. Carbamidomethylation of cysteine residues (+ 57.021 Da) was set as a static modification while oxidation of methionine residues (+ 15.995 Da) was set as a variable modification. Resulting peptides were further filtered to obtain a 1% protein FDR and proteins were collapsed into groups. Reporter ion intensities were adjusted to correct for impurities during synthesis of different TMT reagents according to the manufacturers’ specifications. Lastly, protein quantitative values were column normalized so that the sum of the signal for all protein in each channel was equal to account for sample loading differences and a total sum signal-to-noise of all report ion ions of 100 was required for analysis.

### RNA isolation and microarrays gene expression study—transcriptomics analysis

Dry pellets containing 1 million iPS cells each were suspended in 200 µl of RNA-Later Solution (Life Technologies, Carlsbad, CA, USA) for microarray expression analysis. Cells were stored at − 80 °C. Total RNA was extracted by using the RNeasy Mini Kit (Qiagen, Hilden, Germany), according to the manufacturer's protocol. The contamination of DNA was removed by DNA digestion using RNase-Free DNase Set (Qiagen, Hilden, Germany). RNA concentration was determined by spectrophotometrical measurement using NanoDrop 8000 Spectrophotometer (Life Technologies, Carlsbad, CA, USA). The quality of extracted RNA samples was assessed with Agilent 2200 TapeStation Bioanalyzer (Agilent Technologies, Santa Clara, CA, USA) using RNA Screen Tape Kits or High Sensitivity RNA Screen Tape Kits (Agilent Technologies, Santa Clara, CA, USA). Samples were stored at − 80 °C and selected for further analysis if they had a RNA Integrity Number (RIN) > 8.

Next, a next generation transcriptome-wide gene-level expression profiling was performed using Clariom™ S Assay (Applied Biosystems, Thermo Fisher Scientific, MA, US). In this study, the Affymetric GeneChip® System 3000Dx v.2 platform (Thermo Fisher Scientific, MA, US) consisting of GeneChip® Hybridization Oven, GeneChip® Fluidics Station 450Dx and GeneChip® Scanner 3000Dx with AutoLoader was used.

### Assessment of mitochondrial metabolic activity

In order to validate the results obtained from the proteomic analysis an additional assessment of mitochondrial respiratory chain activity in NSC WFS cells and NSC control cells has been evaluated with the use of Resazurin, as described previously [27]. An aqueous stock solution of Resazurin (Sigma R7017) with a final concentration of 0.5 mM (500 µM) was used. After the removal of the culture medium, the cells were rinsed twice with PBS containing Ca^2+^ and Mg^2+^ ions. Next, 0.5 ml of PBS (Ca^2+^/Mg^2+^) containing 5 mM glucose and 0.5 mM Resazurin was added to each well of the 24-well plate where the desired cells number was previously seeded. Fluorescence measurement was carried out using the Infinite M200Pro reader (Tecan Trading AG, Switzerland) with the Magellan operating software.

### Quantification of protein level by sulforhodamine B (SRB) assay

The protein concentration in each well was determined after the measurements of mitochondrial metabolic activity to standardize this parameter to the protein level in each well of the multi well plate using an SRB assay [28]. After the measurements of mitochondrial metabolic activity, cells were fixed in 1% acetic acid in ice-cold methanol for at least 24 h and the standard SRB procedure was followed. Colorimetric SRB measurement was performed using Infinite M200 plate reader (Tecan Trading AG, Switzerland) with the 595 nm wavelength.

### Evaluation of mitochondrial morphology—transmission electron microscopy (TEM)

In order to perform morphological analysis of mitochondria in NSC WFS cells and control cells, transmission electron microscopy (TEM) was used. Cells were fixed by 2.5% glutaraldehyde and 2% paraformaldehyde (Electron Microscopy Sciences, PA, USA) solution for 1 h in 4 °C. After washing three times for 10 min in 0.1 M cacodylate buffer (BDH Chemicals, VWR, PA, USA). Cells were postfixed in 2% osmium tetroxide (Agar Scientific, Sigma-Aldrich, Germany) for 1 h at room temperature and rinsed three times for 10 min in deionized water. Dehydration was performed by incubating the sample in increasing ethanol concentrations and next in pure propylene oxide. During dehydration cells were stained with 1% uranyl acetate (Serva, Heidelberg, Germany) in 70% ethanol. Finally, cells were embedded in the mixture of propylene oxide (Electron Microscopy Sciences, PA, USA) and Epon resin (Serva, Heidelberg, Germany) then in pure Epon resin. After polymerization in 60 °C, seventy nanometer thick sections were cut using Ultramicrotome (Leica, Vienna, Austria) and collected on TEM copper grids (Ted Pella, CA, USA). Electron micrographs were obtained with Morada camera on a JEM 1400 transmission electron microscope at 80 kV (JEOL Co., Tokio, Japan) in the Laboratory of Electron Microscopy Core Facility, Nencki Institute of Experimental Biology, Polish Academy of Sciences, Warsaw, Poland.

### Validation in publicly available datasets

Dataset for the in silico hypothesis validation—RNA-sequencing data from hippocampi and hypothalami of W*fs1*-deficient mice—was obtained from the publicly accessible database—Gene Expression Omnibus (GEO)—under accession number GSE102625. RNA-seq data analysis was performed using Galaxy platform (https://usegalaxy.org). Reads were preprocessed with Trim Galore! (Galaxy Version 0.6.3) on default settings and mapped to the mm10 genome by using TopHat (Galaxy Version 2.1.1). The read coverage was computed with featureCounts (Galaxy Version 1.6.4 + galaxy1). Finally, the expression values were normalized with TPM normalization method (Galaxy Version 0.4.0) for further analyses. Pathway enrichment analysis was performed with Gene Set Enrichment Analysis (GSEA 4.0.0) platform using REACTOME gene sets (REACTOME_MITOCHONDRIAL_PROTEIN_IMPORT and REACTOME_RESPIRATORY_ELECTRON_TRANSPORT).

### Statistical analysis

Initially, Wolframin expression was compared between WFS and healthy samples, both before and after administration of tunicamycin, using two-sided unpaired student’s t-test and Bonferroni’s correction for multiple hypothesis testing.

Global differences between study groups were investigated with Principal Component Analysis (PCA) and hierarchical clustering (HCL) performed with Multiple Experiment Viewer (MeV 4.8). In two-dimensional PCA plot, groups were manually clustered and annotated. Parameters of HCL included Euclidean distance as distance measure and average linkage algorithm. Based on these analyses, outlier samples were identified and excluded.

For quantitative proteomics data analysis, fold change for each protein in every comparison was calculated and statistical significance was obtained using two-sided unpaired student’s t-test, followed by Benjamini–Hochberg correction for multiple hypothesis testing (FDR). Results were visualised in using the volcano plots.

Gene Set Enrichment Analysis (GSEA) was performed for each comparison of investigated groups with Broad GSEA software (4.0.3) using Reactome 7.0 gene sets collection. Analyses were performed with 1000 permutations (gene set) and default ranking parameters.

GSEA of WFS versus Healthy comparison was visualised as enrichment map in Cytoscape 3.8.0 using the EnrichmentMap 3.3.0 plugin, with overlap coefficient 0.1 and FDR 0.05 as parameters. Network was clustered with AutoAnnotate 1.3.3, manually trimmed and annotated.

Graphical summary of pathways significantly expressed in WFS was created with BioRender.

The ANOVA Kruskal–Wallis test was used for analysis of mitochondrial morphology. Raw data along with respective statistical analysis results were presented in Additional file 1: Table S1.

## Results

**Fig. 1:**
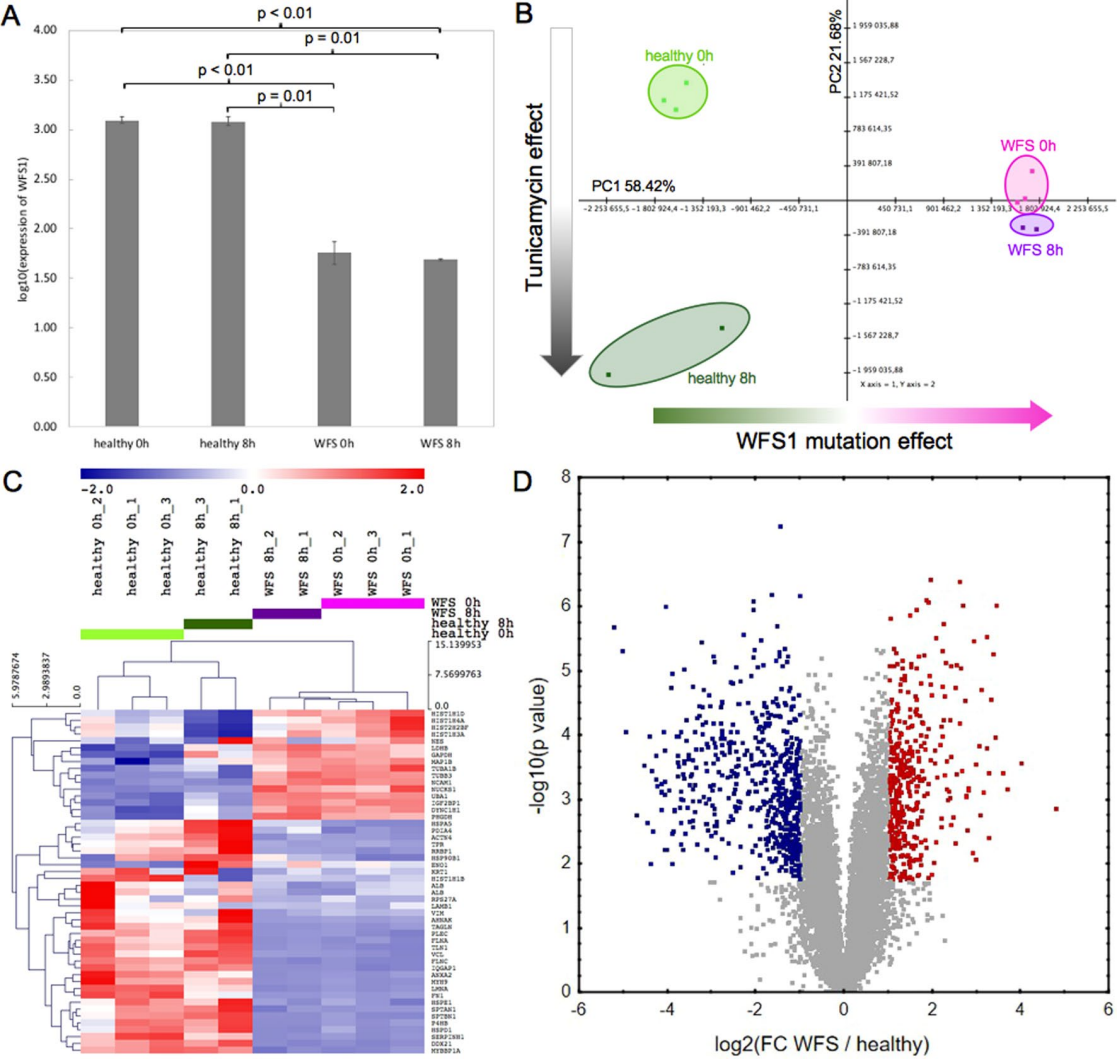
Protein expression in neuronic stem cells from WFS patients and controls before and after tunicamycin administration measured by proteomics experiment. **A** Log_10_ mean abundance of Wolframin across study groups. Whiskers represent standard deviation. One-way ANOVA *p* < 0.0001, *p* values of post-hoc Tukey tests are shown above corresponding groups; **B** Principle Component Analysis plot with annotated study groups. Arrows were added to components 1 and 2 as explanatory due to the observed effects of experimental reactions; **C** Heatmap with hierarchical clusterization performed on 50 proteins with the highest variance. Color scale represents row-standardized protein levels; **D** Volcano plot showing differential expression between WFS and healthy before tunicamycin administration. Significantly (FDR < 0.05) up-regulated (FC > 2) proteins are depicted in red and down-regulated (FDR < 0.05 and FC < 0.5) proteins are depicted in blue

First, we confirmed reduced expression of Wolframin—a product of *WFS1* gene—in NSC WFS cells in comparison to NSC healthy cells, both before and after the ER stress induction (Fig. 1A). After removing one outlier sample (Fig. 1B), a comprehensive proteomic analysis also indicated clusters of specific proteins in NSC cells from WFS patients compared to controls (Fig. 1C). The up- and down-regulated proteins from the study group were presented on volcano plots in reference to the control group before TM administration (Fig. 1D) and other control groups (see Additional file 2: Figure S1).

**Fig. 2:**
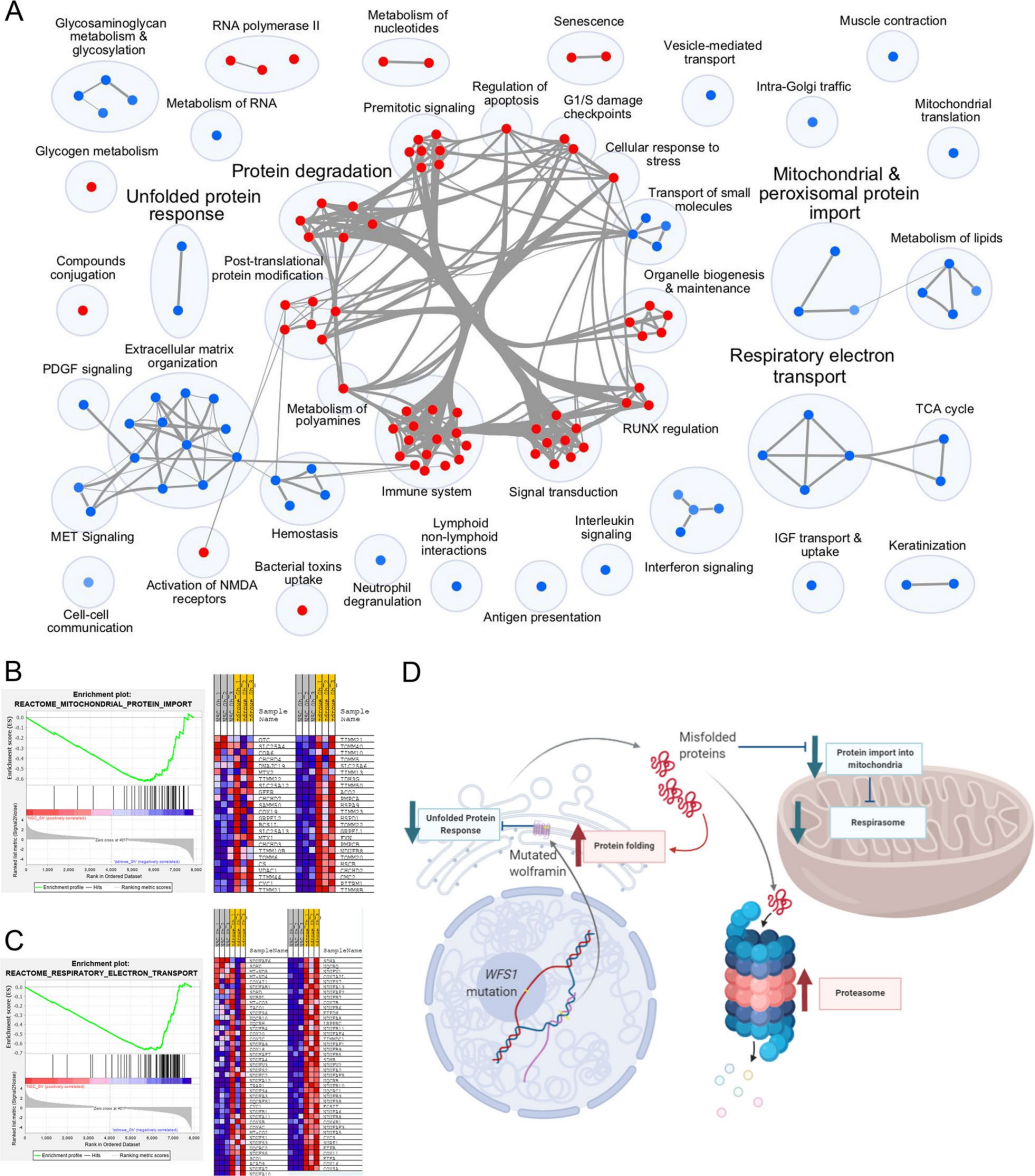
Pathways significantly expressed in WFS patients compared to control. **A** Enrichment map presenting Reactome Gene Sets significant in comparison: WFS versus healthy. Upregulated pathways are shown in red and downregulated ones in blue. **B** Enrichment plot and heatmap of Reactome mitochondrial protein import Gene Set. Heatmap depicts the genes that comprise the pathway with red tiles representing increased expression in WFS versus controls. **C** Enrichment plot and heatmap of Reactome respiratory electron transport Gene Set. **D** Graphical summary of pathways significantly expressed in WFS

Next, specific signal pathways were identified which distinguished NSC WFS cells from healthy ones in proteomic analysis. An enrichment map has been created to show important gene sets relevant for the comparison of WFS to control (Fig. 2A) with particular focus on the Reactome mitochondrial protein import Gene Set (Fig. 2B) and Reactome respiratory electron transport Gene Set (Fig. 2C).

In normal conditions (without induced ER stress), NSC WFS cells showed significantly lower expression of proteins associated with UPR in relation to control (FDR = 0.001). However, the TM administration significantly increased the expression of UPR-related proteins in NSC WFS (FDR = 0.032) (Table 1), which also corresponded to the results of transcriptomic analysis in the WFS cells (FDR = 0.001) (Table 2). Furthermore, in normal conditions WFS NSC cells had significantly higher expression of proteins associated with protein folding in relation to healthy cells (FDR = 0.004) without the significant impact of the TM addition (Table 1). Then, activation of proteasome for protein Gli1 degradation was also observed in proteomic analysis in NSC WFS in relation to controls (FDR = 0.003) and in control cells after TM administration (FDR < 0.001) (Table 1), which in this case was also associated with increased transcription of proteasomal genes (FDR = 0.029) (Table 2).

**Table 1:** Summary of Reactome Gene Sets expression across comparisons in proteomics analysis

Pathway	WFS versus healthy			WFS after tunicamycin versus WFS before			Healthy after tunicamycin versus healthy before		
Reactome unfolded protein response UPR	↓	− 1.97	0.001	↑	2.04	0.032	↑	1.46	0.083
Reactome protein folding	↑	2.09	0.004	↓	− 0.81	0.959	↑	1.41	0.106
Reactome degradation of Gli1 by the proteasome	↑	2.06	0.003	↑	1.22	0.372	↑	3.19	< 0.001
Reactome mitochondrial protein import	↓	− 2.18	< 0.001	↓	− 1.19	0.496	↓	− 2.18	< 0.001
Reactome respiratory electron transport	↓	− 2.59	< 0.001	↓	− 1.63	0.064	↓	− 2.64	< 0.001

*WFS*, Wolfram syndrome; *UPR*, unfolded protein response

**Table 2:** Summary of transcript-level Gene Set Enrichment Analysis across comparisons in transcriptomics analysis

Pathway	WFS versus healthy			WFS after tunicamycin versus WFS before			Healthy after tunicamycin versus healthy before		
Reactome unfolded protein response UPR	↓	− 1.29	0.309	↑	2.25	0.001	↑	1.59	0.125
Reactome protein folding	↓	− 1.49	0.156	↓	− 1.00	0.751	↓	− 1.77	0.034
Reactome degradation of Gli1 by the proteasome	↓	− 1.65	0.092	↓	− 1.68	0.206	↓	− 1.81	0.029
Reactome mitochondrial protein import	↓	− 1.25	0.334	↓	− 1.09	0.750	↓	− 1.11	0.463
Reactome respiratory electron transport	↓	− 1.98	0.022	↓	− 1.63	0.201	↓	− 2.30	< 0.001

Interestingly, in normal conditions (without induced ER stress), NSC WFS cells had significantly lower expression of proteins associated with mitochondrial protein import in relation to control cells (FDR < 0.001) and in healthy cells after TM supply (FDR < 0.001) (Table 1). Finally, this was also associated with a reduction in the respiratory electron transport in NSC WFS cells compared to control (FDR < 0.001) and in healthy NSC cells after TM (FDR < 0.001) observed both in proteomic analysis (Table 1) and transcriptional analysis (Table 2) (FDR = 0.022 and < 0.001, respectively).

A graphical summary of described pathways in WFS patients after the induction of ER stress due to Wolframin deficiency is presented in Fig. 2D.

**Fig. 3:**
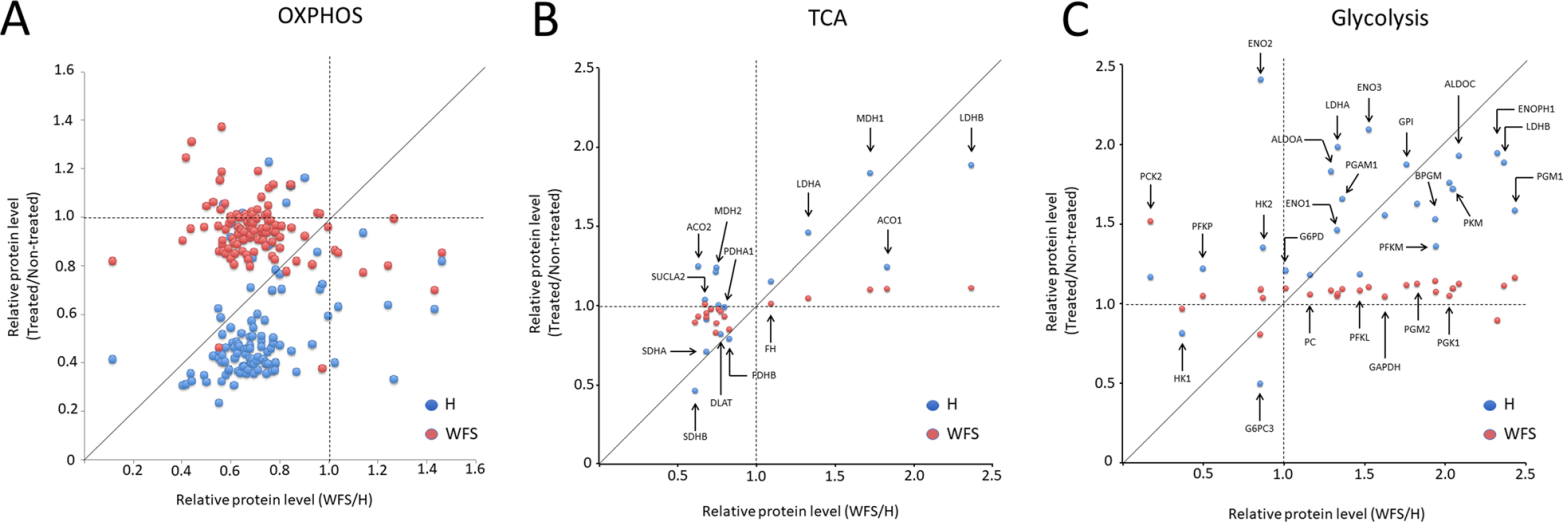
Relative protein level for the individual mitochondrial function elements. WFS samples are shown in red, healthy control (H) in blue. **A** OXPHOS Total, **B** TCA, **C** Glycolysis

**Fig. 4:**
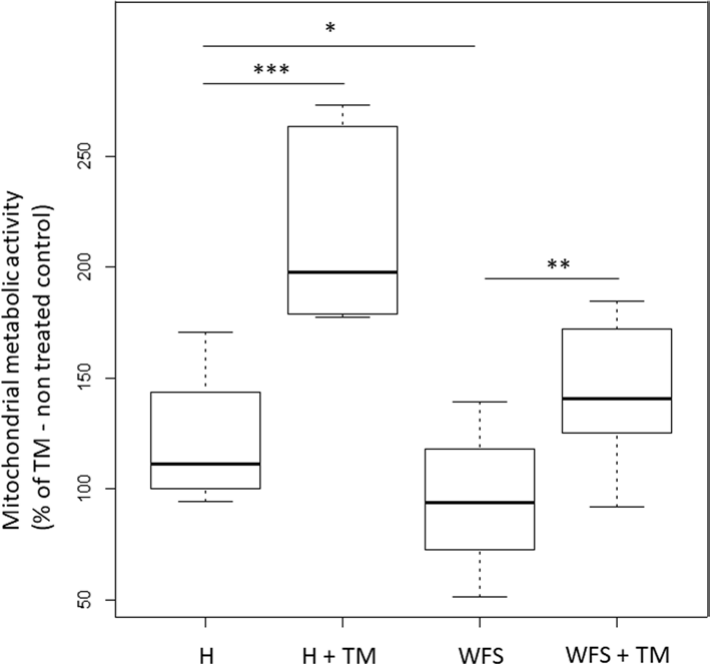
Mitochondrial metabolic activity. Mitochondrial metabolic activity shown as a percentage of healthy (H) TM—non treated cells; (H + TM)—healthy cells treated with TM; (WFS)—patient’ TM—non treated cells; (WFS + TM)—patient’ TM treated cells. TM—tunicamycin; *p < 0.05, **p < 0.01, ***p < 0.001

Moreover, a detailed analysis of the proteins involved in OXPHOS (oxidative phosphorylation) indicated the down-regulation of subunits respiratory chain complexes in NSC WFS cells in comparison to the control cells (Fig. 3A). The level of subunits for individual respiratory chain complexes (complexes I, II, III, IV and V) is shown in Additional file 3: Figure S2. Such alterations are accompanied by up-regulation of both the Krebs cycle (tricarboxylic acid cycle-TCA) and glycolysis-involved proteins in NSC WFS compared to control cells (Fig. 3C–D). The effect of TM administration was not observed in NSC WFS cells, unlike in control cells. This seems to be consistent with the Resazurin assay. Resazurin is a redox reactive compound widely used as reporter agent in assays such as cell viability or metabolic activity due to its fluorometric properties and can be used to visualize mitochondrial activity. The mitochondrial activity in NSC control cells after TM administration was around two times higher than in TM–non treated control cells. On the other hand, TM supply to WFS NSC cells caused only 40% increase (Fig. 4). The basal mitochondrial (metabolic) activity of TM-non treated WFS NSC cells was slightly decreased in comparison to the TM–non treated NSC control cells.

**Fig. 5:**
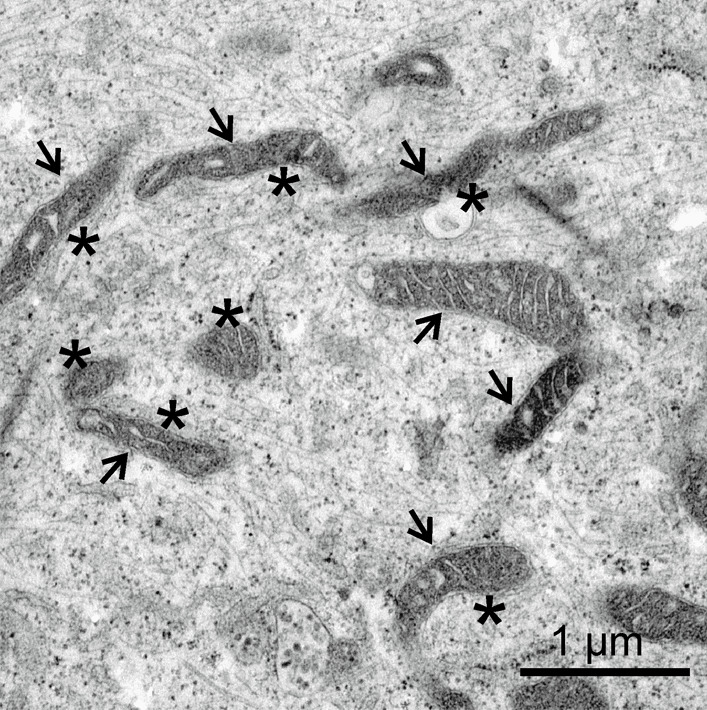
Image from TEM analysis of morphologically altered neural stem cells from WFS patients after tunicamycin administration. Extended mitochondria are marked with arrows, altered mitochondria are marked with stars

The observed mitochondrial dysfunctions were accompanied by abnormalities in mitochondrial morphology in NSC WFS cells after TM administration compared to control NSC with TM, with a predominance of mitochondria with degraded mitochondrial crests (respectively: 82.0% vs. 69.2%; *p* = 0.044) and a tendency to a different percentage of their elongated (60.6% vs. 64.2%; *p* = 0.116, respectively) and circular forms (respectively: 39.4% vs. 35.8%; *p* = 0.116) (Fig. 5).

**Fig. 6:**
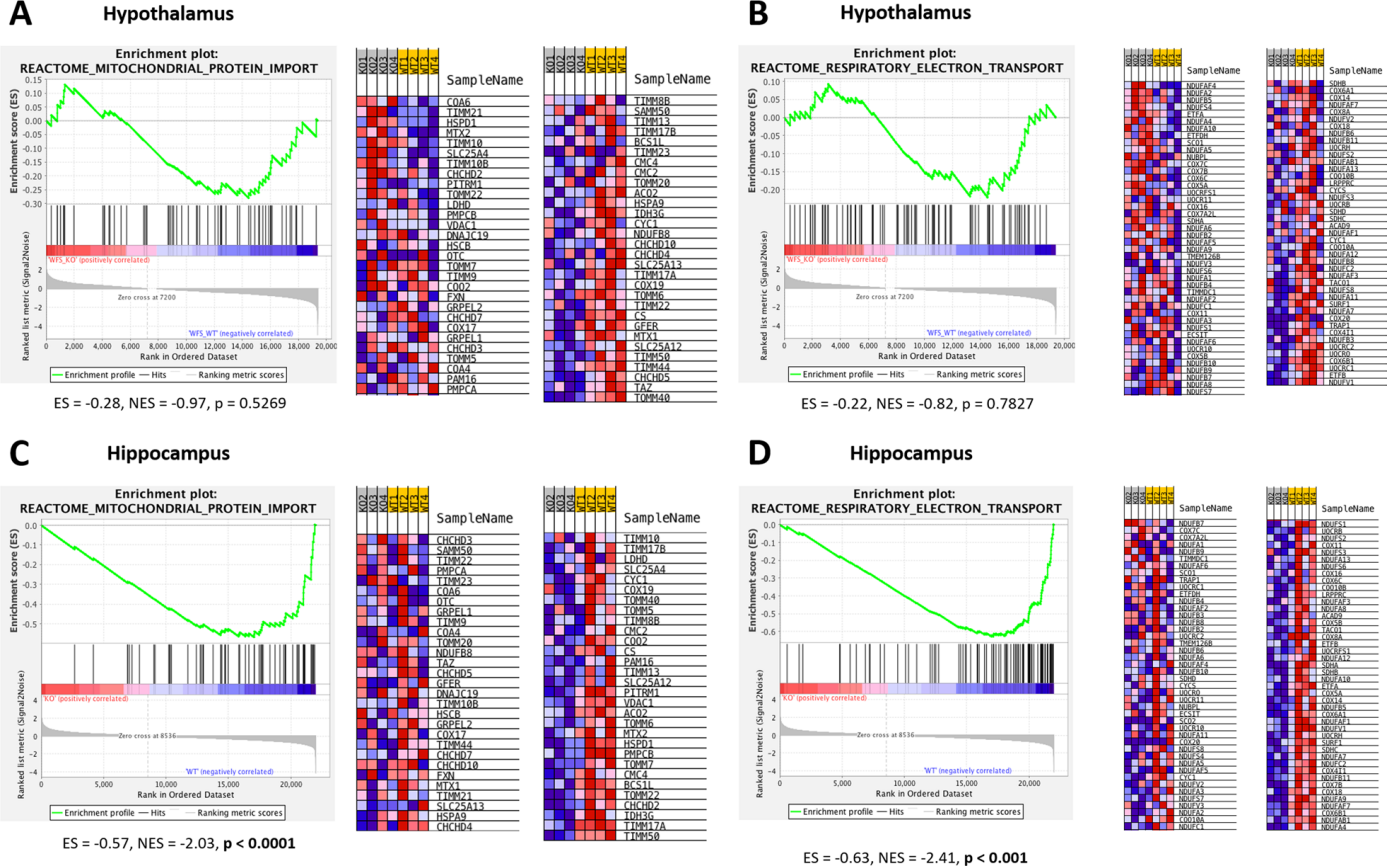
Hypothesis validation on RNA-sequencing mice model data. Genes which contributed to core enrichment are marked on heatmaps with green frames. Heatmaps depict the genes that comprise the pathway with red tiles representing increased expression in WFS versus controls. **A** Enrichment plot and heatmap of Reactome mitochondrial protein import Gene Set performed on transcriptomics data on mice hypothalamic. **B** Enrichment plot and heatmap of Reactome respiratory electron transport performed on transcriptomics data on mice hypothalamic. **C** Enrichment plot and heatmap of Reactome mitochondrial protein import Gene Set performed on transcriptomics data on mice hippocampi. **D** Enrichment plot and heatmap of Reactome respiratory electron transport performed on transcriptomics data on mice hippocampi

In order to validate the presence of mitochondrial abnormalities we accessed publically-available RNA-seq data (GSE102625). This dataset contained results obtained from hippocampi and hypothalami of *Wfs-1* deficient mice. The samples were divided into 2 groups: wild type (WT) and homozygotes for *Wfs1* mutation (KO) to validate in vitro experiment. Using pathway enrichment analysis we showed that that in the hypothalamus of Wfs1^KO/WT^ samples showed down-regulated mitochondrial protein import and respiratory electron transport pathways (Fig. 6A–B). However, the comparisons were not significant (NES =  − 0.97, *p* = 0.5269; NES =  − 0.82, *p* = 0.7827, respectively). In case of hippocampus however both mitochondrial protein import (NES =  − 2.03, *p* < 0.0001) and respiratory electron transport pathways were significantly down-regulated (NES =  − 2.41, *p* < 0.0001) (Fig. 6 C-D) in Wfs1^KO/WT^ animals.

## Discussion

For the first time, proteomic and transcriptomic analyses were performed on a human model of Wolfram syndrome based on iPS cells and NSC line from the skin fibroblasts of patients with WFS and additionally experimental induction of stress ER in such cell lines. This enabled a comprehensive mapping and analysis of the mechanisms occurring in WFS patients from the induction of ER stress due to Wolframin deficiency. Additionally, RNA sequences of hippocampi and hypothalami from *Wfs1*-deficient mice were analyzed.

Our results confirmed a reduced expression of UPR-related proteins and increased expression of proteins associated with protein folding in the analyzed cells in relation to control. Previous studies on pathological mechanisms of WFS highlighted the role of WFS1 as a negative regulator of the UPR-pathway and the significant contribution of hyperactive ATF6 to the activation of apoptosis-promoting genes in the course of WFS [4–6, 29].

We have also observed increased activation of proteasomes for the degradation of Gli1 protein in relation to control. Gli1 is one of the most advanced hedgehog (Hh) signal molecules, which directly regulate the induction of various Hh target genes and are related to development and tissue regeneration, but also to unexpected effects such as cancer or neurodegeneration [30, 31]. Thus, proteasome activation resulting in controlled degradation of proteins with lower molecular weights, including signal proteins with short half-life and abnormally folded proteins seems to be a beneficial effect. Moreover, no similarities can be found with another neurodegenerative disease such as Parkinson's disease (PD), in which the mechanism of inhibiting degradation by proteasome was previously described [32].

However, our proteomic data from NSC cells with *WFS1* mutation showed the most significant decrease in both mitochondrial protein import and respiratory electron transport as compared to control cells. Also, the transcription of genes associated with the mitochondrial respiratory chain was reduced in mutated cell lines, which was consistent with the changes observed by us at the proteomic level. These results, including the analysis of particular subunits of individual respiratory chain complexes, showed severe mitochondrial dysfunction at the level of OXPHOS. In addition, the results of the analysis of RNA-sequencing data from hippocampi of *Wfs1*-deficient mice confirmed a decrease in the pathway associated with mitochondrial protein import, which leads to down-regulation of OXPHOS subunits.

This seems to further support our observations made in the functional analysis of mitochondria, in the Resazurin assay, where we observed much smaller effect of TM in NSC WFS cells in comparison to the TM-treated control cells. All the results indicated the dysfunction of the respiratory chain complex with simultaneous activation of glycolytic processes visualized by the increased level of enzymes involved in this metabolic pathway. To our knowledge, only one proteomic analysis has been performed so far on an animal model of Wolfram syndrome, which included an evaluation of the muscle of *Wfs1*-deficient mice and showed mitochondrial disturbances. A significant increase in the concentration of mitochondrial proteins in the muscle of W*fs1*-deficient mice was noted. Compared to the wild type, the efficiency of ATP synthesis was decreased in the mitochondria of W*fs1*-deficient KO mice due to higher proton leak-dependent respiration, but no difference in OXPHOS-dependent respiration was found [33].

Other studies on rat primary neuronal cultures showed changes in mitochondria regarding their dynamics and selective degradation and inhibition. The authors indicated that a deficiency of the *WFS1* gene could trigger an ER stress cascade that interfered with the IP3 receptor calcium channel and changed calcium homeostasis. This led to increased mitophagy and mitochondrial fusion disorders, which resulted in lower ATP levels and thus inhibited neuronal development [11]. Moreover, these results suggested that a deficiency of *WFS1* might activate the PINK1 and Parkin pathway, participating in mitochondrial movement and dynamics [34, 35]. Other researchers showed that *WFS1* silencing in HEK cells affected gene expression belonging to Parkinson's signaling pathway, indicating the involvement of mitochondria in neurodegenerative disorder development [36].

It is therefore known that interactions between ER stress and mitochondria are crucial for mitochondrial function, regulation of Ca^2+^ homeostasis and apoptosis of affected cells [37, 38]. Thus, some recent studies confirmed the presence of “mitochondria-associated ER membranes” (MAMs), a key player in many neurodegenerative diseases. Their involvement in various neurodegenerative diseases such as Alzheimer disease (AD), PD and Huntington disease (HD) has already been demonstrated [39–42]. Moreover, the participation of MAMs in relation to another subtype of Wolfram syndrome with mutations in the *CISD2* gene—the WFS2 syndrome—was also proved. Despite only mild changes in mitochondrial morphology, the WFS2 patient's fibroblast culture showed a defect in the respiratory chain in complexes I and II, as well as a tendency towards reduced ATP levels [43]. Further studies also indicated a potential functional role of WFS1 in MAMs [11, 40, 44, 45]. A recent study conducted by researchers on skin fibroblasts obtained from patients with WFS1 syndrome did not confirm primary mitochondrial damage or changes in their morphology. However, the authors pointed out that the Ca^2+^ transmission between ER and mitochondria was abnormal and this disorder occurred at the point of contact between the organelles and thus in MAMs [46].

To date, other authors have confirmed that WFS1 can form a complex with a neuronal calcium sensor 1 (NCS1) and inositol 1,4,5-trisphosphate receptor (IP_3_R) promoting Ca^2+^ transfer between ER and mitochondria. In addition, they noted that NCS1 is reduced in *WFS1*-null fibroblasts of WFS1 patients, which decreased the interaction of ER-mitochondria and Ca^2+^ exchange. However, no changes were found in the morphology of mitochondria in *WFS1*-null fibroblasts, as we observed in TEM morphometric analysis in WFS NSC mutant cells. Interestingly, the overexpression of NCS1 in that study not only restored the interaction of ER-mitochondria and Ca^2+^ transfer, but also saved the mitochondrial dysfunction [12].

Thus, our results on NSC cells obtained from skin fibroblasts of WFS1 patients confirm the functional and morphological secondary mitochondrial damage in the course of this disease. The limitation seems to be the use of iPS cells rather than NSC for transcriptomic analysis.

## Conclusions

In summary, it seems that the ER stress in the course of Wolfram syndrome is only the beginning of disorders leading to intensification of changes in the energy center of cells which are mitochondria. This comprehensive assessment is therefore not only of a cognitive nature, but can contribute to the development of future therapeutic intervention in ER-mitochondria cross-talk in patients with Wolfram syndrome.

## Supplementary Information

**Additional file 1: Table S1.** List of genes and proteins generated in omics analyses for the project.

**Additional file 2: Fig. S1.** Global differences between study groups and pairwise differential expressions. **A** Principal Component Analysis with annotated study groups before exclusion of the outlier from healthy 8 h; **B** Healthy 8 h after tunicamycin administration vs healthy before tunicamycin delivery; **C** WFS 8 h after tunicamycin administration vs WFS before tunicamycin administration; **D** WFS before tunicamycin delivery vs healthy 8 h after tunicamycin administration; **E** WFS 8 h after tunicamycin delivery vs healthy 8 h after tunicamycin administration. Significantly (FDR < 0.05) up-regulated (FC > 2) proteins are depicted in red and down-regulated (FC < 0.5) proteins are depicted in blue.

**Additional file 3: Fig. S2**. Relative protein level for specific respiratory chain complexes. WFS samples are shown in red, healthy control (H) in blue. **A** CI of respiratory chain, **B** CII of respiratory chain, **C** CIII of respiratory chain, **D** CIV of respiratory chain, **E** CV of respiratory chain.

## Data Availability

The data analyzed during the current study are available from the corresponding author on request.

## Abbreviations

AD: Alzheimer disease
ATF6: Activating transcription factor 6
FDR: False discovery rate
GEO: Gene Expression Omnibus
GSEA: Gene Set Enrichment Analysis
HCL: Hierarchical clustering
Hh: Hedgehog
HD: Huntington disease
iPS: Induced pluripotent stem cells
IRE1: Requiring inositol factor 1
IDA: Intelligent data acquisition
LC: Liquid chromatography
MAMs: Mitochondria-associated ER membranes
MLPA: Multiplex ligation-dependent probe amplification
MS: Mass spectrometry
NSC: Neural stem cells
OXPHOS: Oxidative phosphorylation
PD: Parkinson disease
PCA: Principal Component Analysis
PERK: Protein kinase RNA-like ER kinase
RTS: Real-time searching
SRB: Sulforhodamine B
TCA: Tricarboxylic acid cycle
TEM: Transmission electron microscopy
TH: Tyrosine hydroxylase
UPR: Unfolded protein response
WFS: Wolfram syndrome
XBP1: X-box binding protein 1
