# Supplementary figures and images for: Multiomic analysis on human cell model of wolfram syndrome reveals changes in mitochondrial morphology and function
Source: Cell Commun Signal. 2021 Nov 20;19:116. doi: 10.1186/s12964-021-00791-2 (PMC8605533; doi:10.1186/s12964-021-00791-2)

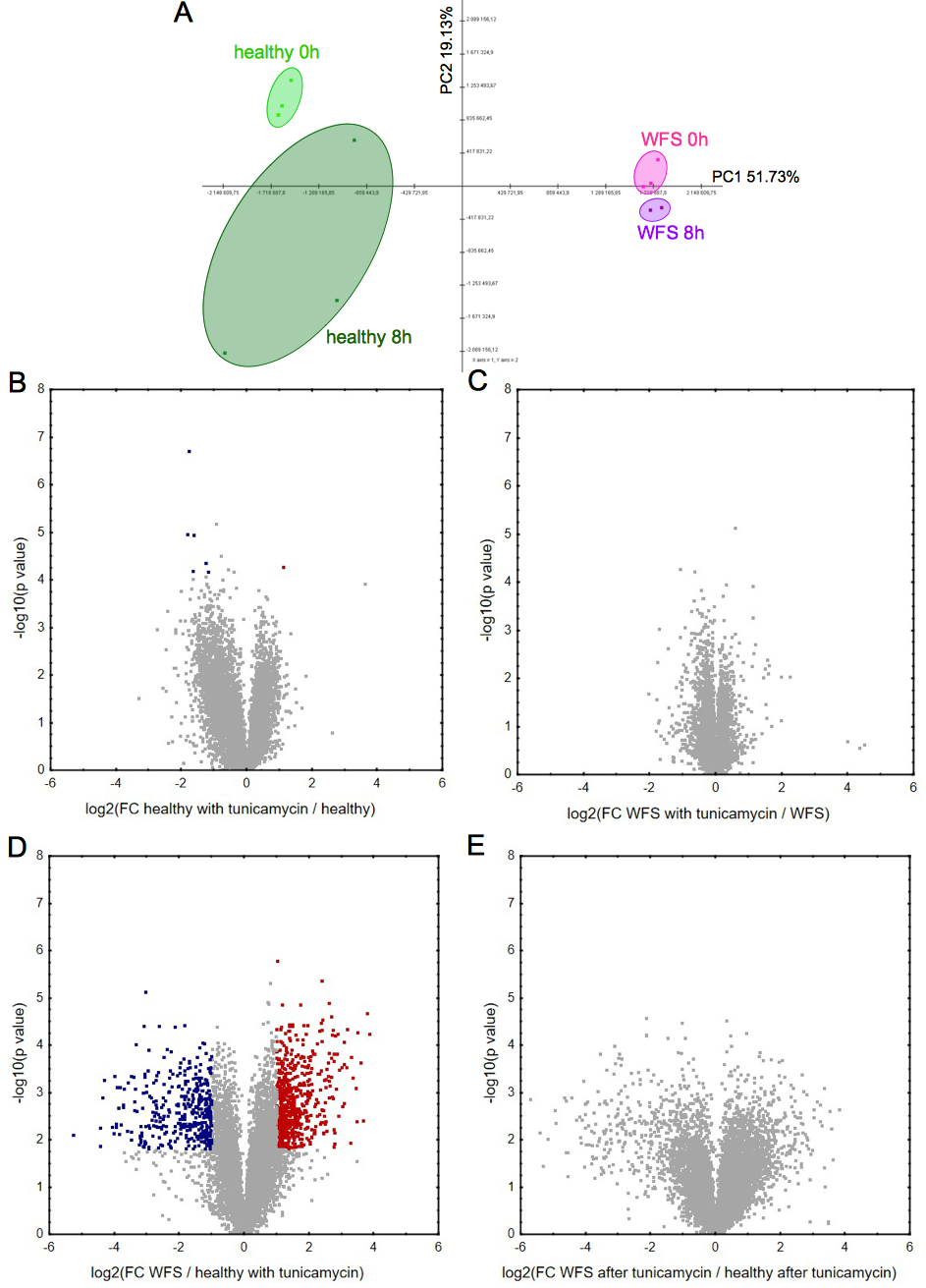

Supplement: Supplementary file 3 — Additional file 2: Fig. S1. Global differences between study groups and pairwise differential expressions. A Principal Component Analysis with annotated study groups before exclusion of the outlier from healthy 8 h; B Healthy 8 h after tunicamycin administration vs healthy before tunicamycin delivery; C WFS 8 h after tunicamycin administration vs WFS before tunicamycin administration; D WFS before tunicamycin delivery vs healthy 8 h after tunicamycin administration; E WFS 8 h after tunicamycin delivery vs healthy 8 h after tunicamycin administration. Significantly (FDR < 0.05) up-regulated (FC > 2) proteins are depicted in red and down-regulated (FC < 0.5) proteins are depicted in blue. [file 12964_2021_791_MOESM3_ESM.tif]

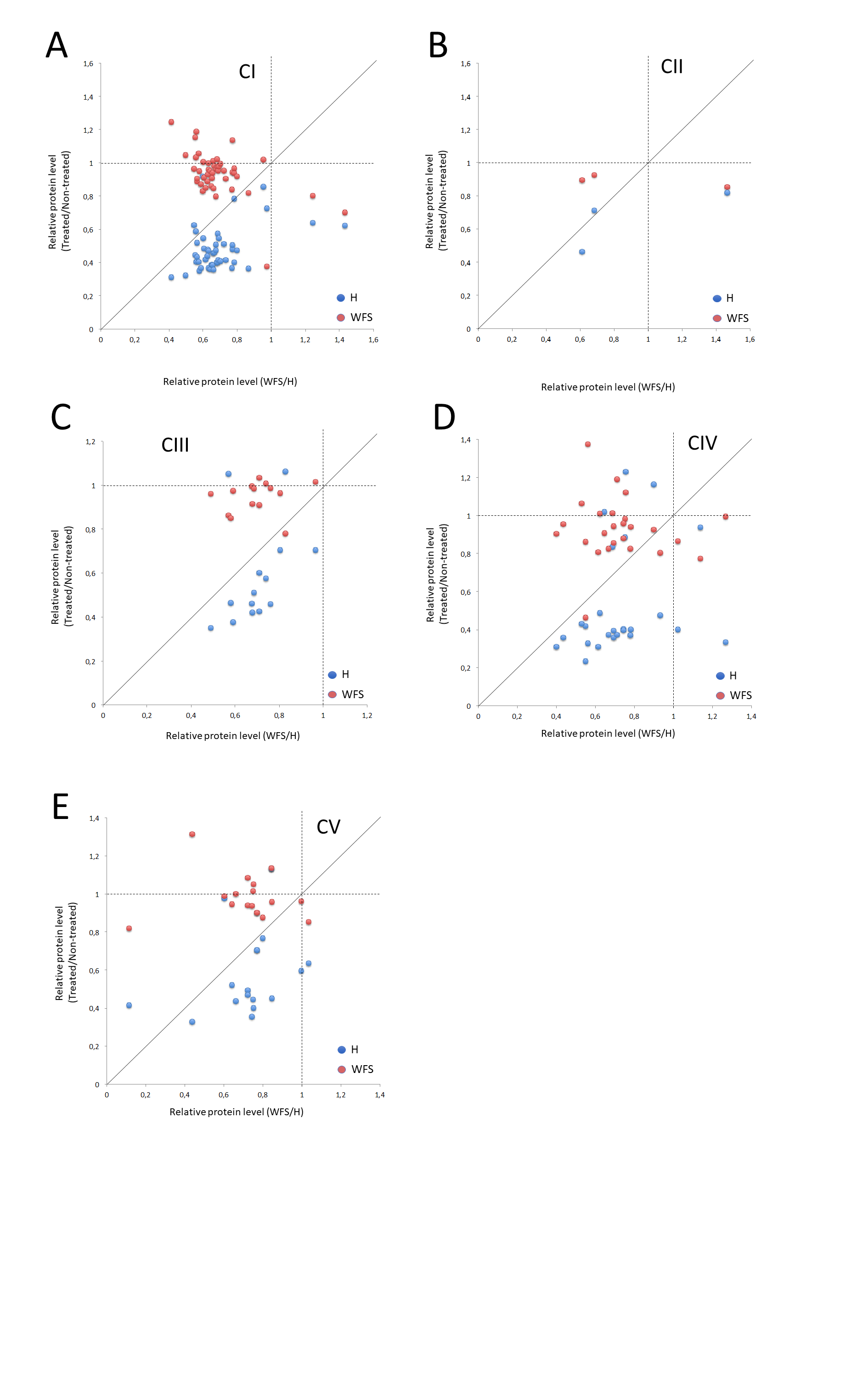

Supplement: Supplementary file 4 — Additional file 3: Fig. S2. Relative protein level for specific respiratory chain complexes. WFS samples are shown in red, healthy control (H) in blue. A CI of respiratory chain, B CII of respiratory chain, C CIII of respiratory chain, D CIV of respiratory chain, E CV of respiratory chain. [file 12964_2021_791_MOESM4_ESM.tif]
